# A Computer-Interpretable Guideline for COVID-19: Rapid Development and Dissemination

**DOI:** 10.2196/21628

**Published:** 2020-10-01

**Authors:** Shan Nan, Tianhua Tang, Hongshuo Feng, Yijie Wang, Mengyang Li, Xudong Lu, Huilong Duan

**Affiliations:** 1 College of Biomedical Engineering and Instrumental Science Zhejiang University Hangzhou China; 2 Information Systems Industrial Engineering & Innovation Sciences Technical University of Eindhoven Eindhoven Netherlands; 3 Hangzhou Vico Software Cooperation Hangzhou China

**Keywords:** COVID-19, guideline, CDSS, openEHR, Guideline Definition Language, development, dissemination, electronic health record, algorithm

## Abstract

**Background:**

COVID-19 is a global pandemic that is affecting more than 200 countries worldwide. Efficient diagnosis and treatment are crucial to combat the disease. Computer-interpretable guidelines (CIGs) can aid the broad global adoption of evidence-based diagnosis and treatment knowledge. However, currently, no internationally shareable CIG exists.

**Objective:**

The aim of this study was to establish a rapid CIG development and dissemination approach and apply it to develop a shareable CIG for COVID-19.

**Methods:**

A 6-step rapid CIG development and dissemination approach was designed and applied. Processes, roles, and deliverable artifacts were specified in this approach to eliminate ambiguities during development of the CIG. The Guideline Definition Language (GDL) was used to capture the clinical rules. A CIG for COVID-19 was developed by translating, interpreting, annotating, extracting, and formalizing the Chinese COVID-19 diagnosis and treatment guideline. A prototype application was implemented to validate the CIG.

**Results:**

We used 27 archetypes for the COVID-19 guideline. We developed 18 GDL rules to cover the diagnosis and treatment suggestion algorithms in the narrative guideline. The CIG was further translated to object data model and Drools rules to facilitate its use by people who do not employ the non-openEHR archetype. The prototype application validated the correctness of the CIG with a public data set. Both the GDL rules and Drools rules have been disseminated on GitHub.

**Conclusions:**

Our rapid CIG development and dissemination approach accelerated the pace of COVID-19 CIG development. A validated COVID-19 CIG is now available to the public.

## Introduction

COVID-19 is a global pandemic that is affecting over 200 countries and territories worldwide [1]. As of June 2020, 8,690,140 cases of COVID-19 have been diagnosed, and 461,274 deaths from the disease have been reported [2]. Medical resources, especially intensive care resources, have been drained by the COVID-19 pandemic in both developed and developing countries [3,4]. Proper prevention, efficient diagnosis, and effective treatment based on established evidence are crucial to save patients, ease the burden of medical workers, and accelerate eradication of the disease [5]. Unfortunately, the perception and knowledge of COVID-19 diagnosis and treatment among caregivers are still at low levels, which significantly hinders the pace of managing the disease [6].

Information technology is crucial for combating the COVID-19 pandemic [7,8]. Many efforts have already been contributed to estimate the trend of the pandemic at national or global levels [9-12], predict the prognosis of an individual patient [13,14], visualize and track reported cases of COVID-19 in real time [15], assist diagnosis based on chest computed tomography images [16], provide telemedicine for chronic disease patients [17], improve caregivers’ work efficiency [18-20], and survey the public attitude and response towards COVID-19 [21,22]. Some electronic medical record (EMR) system vendors have pushed out updates to their software to help caregivers detect potential patients with COVID-19 [23]. However, to our best knowledge, efforts in this line supporting evidence-based COVID-19 diagnosis and treatment are limited. Particularly, a publicly available computer-interpretable guideline (CIG) for COVID-19 has not been reported. Such a CIG could accelerate the wide and rapid adoption of evidence-based diagnosis and treatment guidelines.

The lack of a CIG is unsurprising if one considers the enormous challenges of developing a shareable CIG for COVID-19 in a limited timeframe. Sharing CIGs among different organizations involves many difficulties. The use of a site-specific data model (known as the “curly braces problem”) in a CIG limits it to a specific clinical site [24]. Due to different output formats, it is challenging to integrate a CIG into various EMRs.

Developing a CIG is a time-consuming and resource-dependent process that requires a group of informaticists and medical specialists to work together closely for a considerable period of time because they must engage in a significant number of discussions to eliminate ambiguity and misunderstanding of the narrative guideline during development [25,26]. In the conventional approach, CIG development is broken down into several phases, and the input and output artifacts in each phase are defined at a conceptual level. However, a clear specification of these artifacts has not yet been established, and the roles who should take part in each phase are unclear. Moreover, it is not possible to apply the conventional CIG development approach to COVID-19 because frequent face-to-face discussions are impractical during the pandemic. Few local medical specialists are available to take part in CIG development because these resources are currently scarce [3]. Even informaticists can no longer meet face-to-face in many countries because of local lockdown policies.

An approach that can standardize the input and output of CIGs and leverage existing resources would be helpful. The openEHR standard is a potential solution. From the technology point of view, openEHR aims to facilitate interoperability between information systems [27]. The openEHR archetype provides a standard information model that can be shared among organizations to avoid the “curly braces problem” [24]. From the domain knowledge perspective, openEHR aims to bring informaticists and medical specialists together. Specifically, openEHR uses an archetype to capture detailed and domain-specific clinical concepts that are modeled by clinical specialists [28]. The Guideline Definition Language (GDL) was proposed by the openEHR community to facilitate the use of openEHR archetypes to author CIGs [29]. Recently, GDL was upgraded to its second major version, known as GDL2 [29]. GDL improves the shareability of encoded CIGs among organizations across borders [30,31]. However, there is still a gap between interpreting a narrative guideline and using GDL to author a CIG, especially considering the current difficulties of efficient communication. A specification for informaticists to use GDL to rapidly capture narrative guideline knowledge is urgently required.

This paper proposes a rapid CIG development and dissemination approach using GDL. A sharable CIG enabling automatic diagnosis and treatment of COVID-19 has been developed and disseminated by applying the proposed approach. A prototype application has been developed and validated with public patient data to demonstrate the use of the CIG.

## Methods

Referring to the CIG authoring approach proposed by Zhou et al [26], we designed a rapid CIG development approach that parallelizes data modeling and rule editing. In addition to the original approach, the output of each step was specified to eliminate ambiguities between different participants. Then, we reported the detailed process of applying the approach to develop a CIG for COVID-19 based on the seventh edition of the Chinese COVID-19 Diagnosis and Treatment Plan.

### Design of the Rapid CIG Development Approach

Zhou et al [26] reported a CIG authoring approach with six steps, including (1) create a knowledge specification, (2) integrate with terminology, (3) author rules, (4) test rules, (5) publish rules, and (6) generate reports. These six steps should be carried out sequentially, and key artifacts are produced in each step. Two aspects of this approach should be optimized to support the rapid development of CIGs. First, among these six steps, the first three steps are time-consuming and dependent on medical resources. Second, although key artifacts are categorized in the approach, their contents are not specified, which may still cause ambiguities.

**Figure 1:**
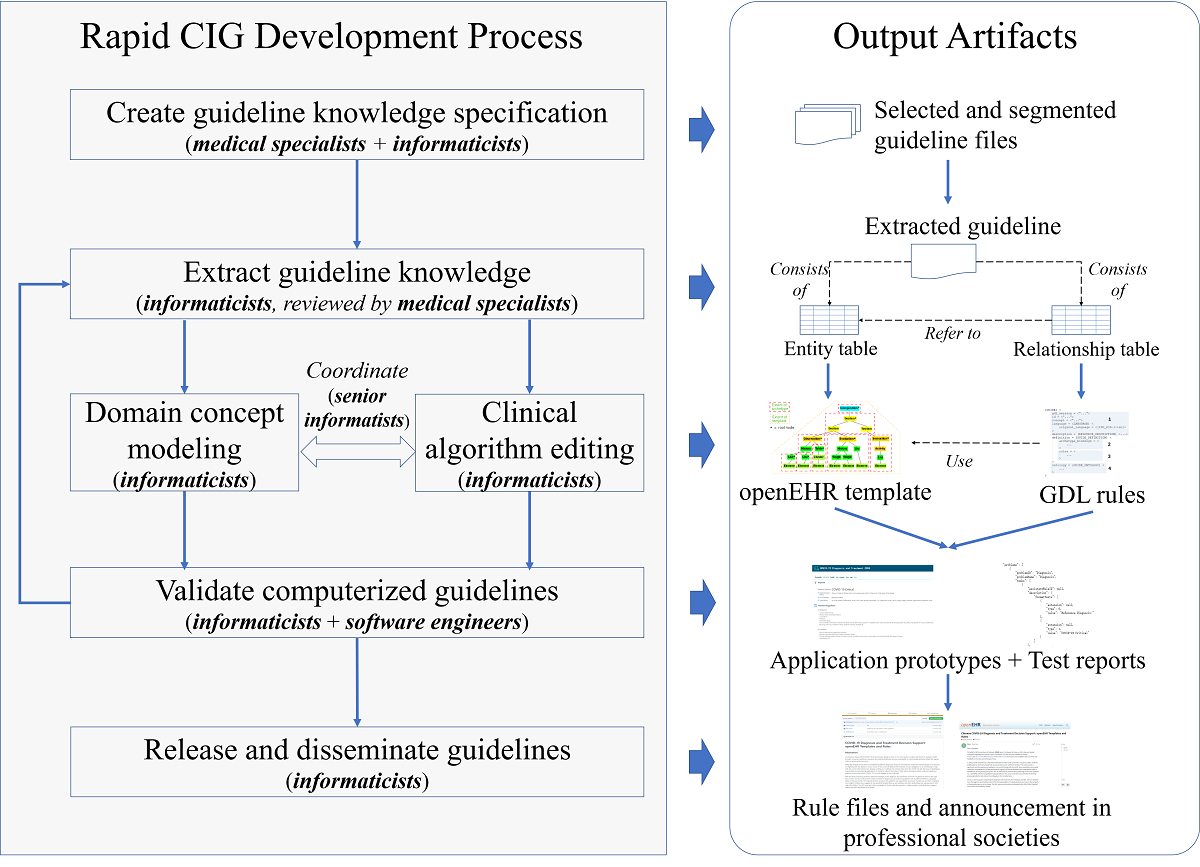
The scheme of the rapid CIG development approach. CIG: computer-interpretable guideline.

In this section, we propose a rapid approach to develop and disseminate CIGs by solving these two problems. The key steps, participants, and output artifacts of each step are specified in our approach. The rapid CIG development and dissemination approach contains six steps (see Figure 1): (1) create guideline knowledge specifications, (2) extract guideline knowledge, (3) model the domain concept, (4) edit the clinical algorithm, (5) validate the computerized guideline, and (6) release and disseminate the guideline. The approach is explained in detail as follows.

#### Step 1: Create Guideline Knowledge Specifications

In this step, a joint CIG development team consisting of both medical specialists and informaticists must be established. Medical specialists provide clinical requirements for decision support. According to the requirements, the informaticists select related guidelines, read through each narrative guideline, segment interesting sections in the guidelines, and finally confirm their selections with medical specialists. Selected and segmented human-readable guideline files are organized as the output of this step.

#### Step 2: Extract Guideline Knowledge

This step bridges the human-readable narrative guideline and the CIG by breaking narrative text into entities and relations. Informaticists read through the narrative guideline and break the guideline into small logic blocks, which can be represented by production rules. Each block contains a left-hand side representing the conditions and a right-hand side representing the consequent actions. These relationships are collected as a relationship table and delivered as an artifact. Both the left-hand side and right-hand side are further broken down from phrases into individual terms. Entities are marked up and extracted from those terms to form an entity table, which is also an artifact. The extraction results must be reviewed by medical specialists to ensure their correctness.

#### Steps 3 and 4: Model the Domain Concept Modeling and Edit the Clinical Algorithm

These steps are performed concurrently and in collaboration by two groups of informaticists to accelerate the development pace. This process should be coordinated by senior informaticists to ensure consistency in both groups. Domain concept modeling refers to the openEHR template development process. Based on the aforementioned entity table, informaticists search the openEHR archetype repository and select suitable archetypes that best represent the entities. An archetype should be created if there is no appropriate archetype for a specific entity. An openEHR template is developed to organize these archetypes. The detailed approach of openEHR template modeling is described elsewhere [32]. In the clinical algorithm editing step, another group of informaticists translates the relationship table into GDL rules. The translation is straightforward because GDL follows the general structure of production rules. When editing the clinical algorithms, the domain concept model is needed as the input. At the same time, the clinical algorithm editing raises domain concept requirements that must be created or refined. This bidirectional dependence must be coordinated by senior informaticists.

#### Step 5: Validate the Computerized Guideline

In this step, the CIG is validated with clinical data. Guideline authoring tools usually contain a CIG validation module, which receives data from manual input by informaticists. The CIG can be further validated by implementing clinical decision support systems (CDSSs). This work requires the involvement of software engineers. If the validation results have any inconsistency with the original narrative guideline, the whole process from guideline extraction to authoring is reviewed.

#### Step 6: Release and Disseminate the Guideline

Finally, the well-developed guideline should be made publicly available by informaticists. Code-sharing platforms such as GitHub and forums of professional societies are good options for disseminating these guidelines.

**Figure 2:**
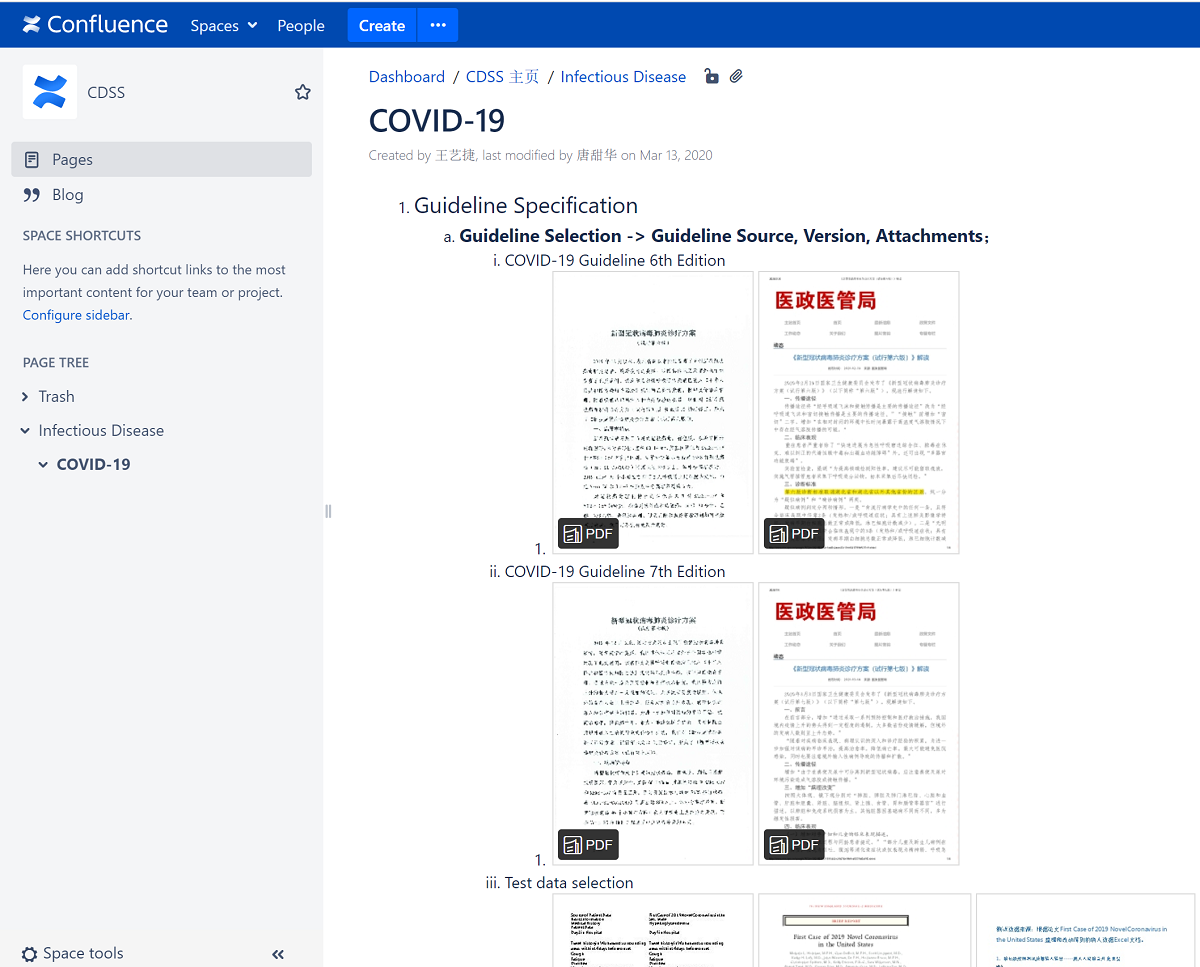
Screenshot of the Confluence knowledge platform configured for the proposed approach.

To manage the proposed 6-step approach, especially considering online collaboration, we used the Atlassian Confluence team collaboration platform [33] as a tool to coordinate the work (see Figure 2). The six steps were elicited on the platform. Each participant was requested to submit their outcome artifacts on the platform. Version controls were implemented to track the history of documents. Archetypes were searched in the openEHR Clinical Knowledge Manager (CKM) [34]. The GDL rules were edited using the GDL2 Editor in docker [35].

### COVID-19 CIG Development

In this section, we report the detailed process of rapid development of a CIG for COVID-19 based on the seventh edition of the COVID-19 Diagnosis and Treatment Plan published by the National Health Commission of the People’s Republic of China (NHC) [36]. An English translation was used as a reference [37].

#### Create Guideline Knowledge Specifications

The Chinese COVID-19 guideline was published by the NHC to share the latest evidence-based best practices regarding COVID-19 diagnosis and treatment and to nationally standardize caregiver practices.

The seventh edition of the Chinese COVID-19 Diagnosis and Treatment Plan consists of 13 sections. Sections 1 to 4 provide general introductions to the pathogen, epidemiology, pathology, and patient features of SARS-CoV-2. Then, in sections 5 to 8, the diagnostic criteria are discussed in detail. In Section 9, guidelines are defined as to how cases should be reported. In Section 10, treatment plans are introduced. From Section 11 to Section 13, discharge criteria, transportation, and in-hospital infection are briefly discussed. The CIG development team spent three days reading and discussing the guideline. Because our primary goal was to develop an executable guideline, the CIG development team jointly decided to select the diagnosis and treatment sections (ie, sections 5 to 8 and sections 10 and 11). The traditional Chinese medicine section was not included in the treatment suggestions because it is not available in other countries. Both the original copy published by the NHC and the English version translated by a public society were used in the next steps.

#### Extract Guideline Knowledge

One informaticist (TT) read the guideline and split the sentences into a spreadsheet (Multimedia Appendix 1). Four days were spent on this task. A color system was used in the splitting: blue indicated that the sentence was related to diagnosis or treatment; black indicated that the sentence was not suitable for translation to a clinical rule; and red indicated that the sentence contained vague content and required later consultation with medical specialists. The document was uploaded to the Confluence platform in a timely fashion. Each update was double-checked by two other informaticists (SN and HF).

Then, TT annotated the entities in the text and extracted them into another spreadsheet (Multimedia Appendix 2). Repeated entities were merged. TT uploaded the document to Confluence, and two other informaticists (HF and ML) double-checked the annotation and the extracted results. The final extracted results were reviewed and confirmed by an external medical specialist. Two additional days were spent on this task. In total, this step took six days.

#### Model the Domain Concept

Based on the entity table developed in the previous step, ML and HF mapped the listed entities to the openEHR concepts. While mapping, the openEHR template was expanded accordingly. The mapping was checked by a senior informaticist (SN) and an external openEHR expert. A group of archetypes in the format of archetype definition language (ADL) files were exported from the CKM. The detailed archetype searching and template development process is reported elsewhere [32]. Ten days were required for the domain concept modeling because the selection of proper archetypes required confirmation by the external openEHR expert. Three rounds of teleconferences were held to finalize the domain concept model.

#### Author the Clinical Algorithm

Sentences in the narrative guideline were broken down into the left-hand side and right-hand side blocks by TT in the guideline extraction step. In this step, HF used the GDL2 Editor to encode the clinical algorithms in GDL.

ADL files describing the COVID-19 data requirements were imported into the GDL2 Editor. Following the structure of the extracted guideline file, HF translated each left-hand side and right-hand side pair to a GDL rule in a *when-then* format.

The GDL rules were checked and confirmed by SN. SN and HF took part in both the domain concept modeling and clinical algorithm authoring. They bridged the two groups of informaticists and lowered the communication cost. The clinical algorithm authoring was performed at the same time as the domain concept modeling. Once a part of the domain concept model was finalized, the related GDL rules were created accordingly. The entire authoring process was synchronized with the domain concept modeling step.

#### Validate the Computerized Guideline

The CIG developed in the previous step was validated both by GDL2 Editor and a prototype CDSS for COVID-19. Our research team previously developed a configurable CDSS platform named Tracebook to develop CDSS applications rapidly [38]. In this study, we used the Tracebook platform to configure a fast prototype of a COVID-19 CDSS. Because there are no open-source or openly available GDL2 execution engines, we chose the Drools rule engine to execute the clinical rules [39]. Mapping was required between GDL2 and Drools at both the data model level and the language level. The mapping rule and mapping specification were defined jointly by SN, TT, and HF (Multimedia Appendix 2). Then, the mapping was performed manually by TT. An additional Drools rule for the user interface presentation was also developed. The additional rule mapping and system development were performed over five days.

Test patient data were adopted from patient case report published in a medical journal [40]. The patient was a 35-year-old man who had cough and fever symptoms and had recently traveled to Wuhan, China. The patient’s demographic information, history, and observations were captured from the publication and entered into both the GDL2 Editor guideline validation module and our own CDSS. The output was compared with both the guideline and the reported diagnosis and prescription. Inconsistencies between these three outputs were reported to external medical specialists, and the CIG was reviewed.

#### Release and Disseminate the Guideline

Archetypes in ADL file format and the GDL rules in GDL2 format were exported from their editors, packaged together, and committed to GitHub [41]. Java data models and Drools rules were also committed to GitHub to benefit people who do not use openEHR. Then, the dissemination was reported on the openEHR disclosure forum [42].

## Results

In this section, we illustrate our GDL COVID-19 guideline model and the validation results.

### The Computerized COVID-19 Guideline

#### Domain Concept Model

The domain concept model is illustrated in detail in Table 1.

A total of 27 archetypes were used for the COVID-19 CIG, among which 26 were directly acquired from the CKM and 1 (openEHR-EHR-CLUSTER.imaging_result-COVID_19.v0) was acquired from the CKM and modified for the COVID-19 CDSS. These 27 archetypes were sorted into 9 categories: demographic, history, medical record, exam, vital sign, laboratory test, symptom, diagnosis, and order. The organization of the Java data models followed the concept categories.

**Table 1:** List of concept categories, used openEHR archetypes, and their associated models.

Concept category	openEHR archetypes	Object data model
Demographic	openEHR-EHR-OBSERVATION.age.v0	PatientInfo
History	openEHR-EHR-OBSERVATION.exposure_assessment.v0	EpidemicHistory
Medical record	openEHR-EHR-OBSERVATION.pf_ratio.v0 openEHR-EHR-OBSERVATION.story.v1	MedicalRecord
Exam	openEHR-EHR-CLUSTER.imaging_finding.v0 openEHR-EHR-CLUSTER.imaging_result-COVID_19.v0 openEHR-EHR-OBSERVATION.imaging_exam_result.v0	ImgExamResult
Vital sign	openEHR-EHR-CLUSTER.inspired_oxygen.v1 openEHR-EHR-CLUSTER.level_of_exertion.v0 openEHR-EHR-CLUSTER.problem_qualifier.v1 openEHR-EHR-OBSERVATION.body_temperature.v2 openEHR-EHR-OBSERVATION.pulse_oximetry.v1 openEHR-EHR-OBSERVATION.respiration.v2	PhysicalSign
Laboratory test	openEHR-EHR-CLUSTER.specimen.v0 openEHR-EHR-CLUSTER.laboratory_test_analyte.v1 openEHR-EHR-OBSERVATION.laboratory_test_result.v1	LabTestResult
Symptom	openEHR-EHR-CLUSTER.symptom_sign.v1 openEHR-EHR-COMPOSITION.encounter.v1 openEHR-EHR-OBSERVATION.symptom_sign_screening.v0 openEHR-EHR-OBSERVATION.condition_screening.v0	Symptom
Diagnosis	openEHR-EHR-EVALUATION.differential_diagnoses.v0 openEHR-EHR-EVALUATION.health_risk.v1 openEHR-EHR-EVALUATION.problem_diagnosis.v1	Diagnosis
Order	openEHR-EHR-EVALUATION.recommendation.v1 openEHR-EHR-INSTRUCTION.medication_order.v2 openEHR-EHR-INSTRUCTION.therapeutic_order.v0 openEHR-EHR-OBSERVATION.management_screening.v0	Order

#### Algorithm Model

The COVID-19 diagnosis and treatment rules are listed in Table 2. 

Sections 5 to 8 and sections 10 and 11 of the Chinese COVID-19 Diagnosis and Treatment Plan were encoded in both GDL and Drools. These rules support diagnosis, classification, early warning, treatment, and discharge for caregivers.

For sections 5, 10, and 11, there are multiple GDL rules for one section. This is because GDL2 Editor now only allows one rule in a file, whereas these sections contain several rules. This limitation does not exist in Drools; therefore, we merged the rules for one purpose into one Drools rule file.

**Table 2:** List of created GDL and Drools rules for the associated sections of the Chinese COVID-19 Diagnosis and Treatment Plan.

Section	GDL^a^ rules	Drools rule
5. Diagnostic Criteria	COVID_Confirmed_Diagnosis.v0.gdl2 COVID_Lymphocyte_count.v0.gdl2 COVID_Nucleic_acid_test_result.v0.gdl2 COVID_White_blood_cell_count.v0.gdl2 COVID_White_cell_count.v0.gdl2	Diagnosis_Confirmed
6. Clinical Classification	COVID_Classfication.v0.gdl2	Classification
7. Clinical Warning Sign	COVID_Clinical_Warning.v0.gdl2	Clinical_Warning
8. Differential Diagnosis	COVID_Suspected_Diagnosis.v0.gdl2	Diagnosis_Suspected
10. Treatment	COVID_Blood_Purification_Treatment.v0.gdl2 COVID_Circulation_support_Treatment.v0.gdl2 COVID_Continuous_Renal_Replacement_Therapy.v0.gdl2 COVID_Convalescent_plasma_Treatment.v0.gdl2 COVID_General_Treatment.v0.gdl2 COVID_Immunotherapy.v0.gdl2 COVID_Other_Treatment.v0.gdl2 COVID_Respiratory_support_Treatment.v0.gdl2	Treatment_Modern
11. Discharge	COVID_Body_Temperature_Monitor.v0.gdl2 COVID_Out_Hospital.v0.gdl2	Discharge

^a^GDL: Guideline Definition Language.

### Validation of the Guideline

**Figure 3:**
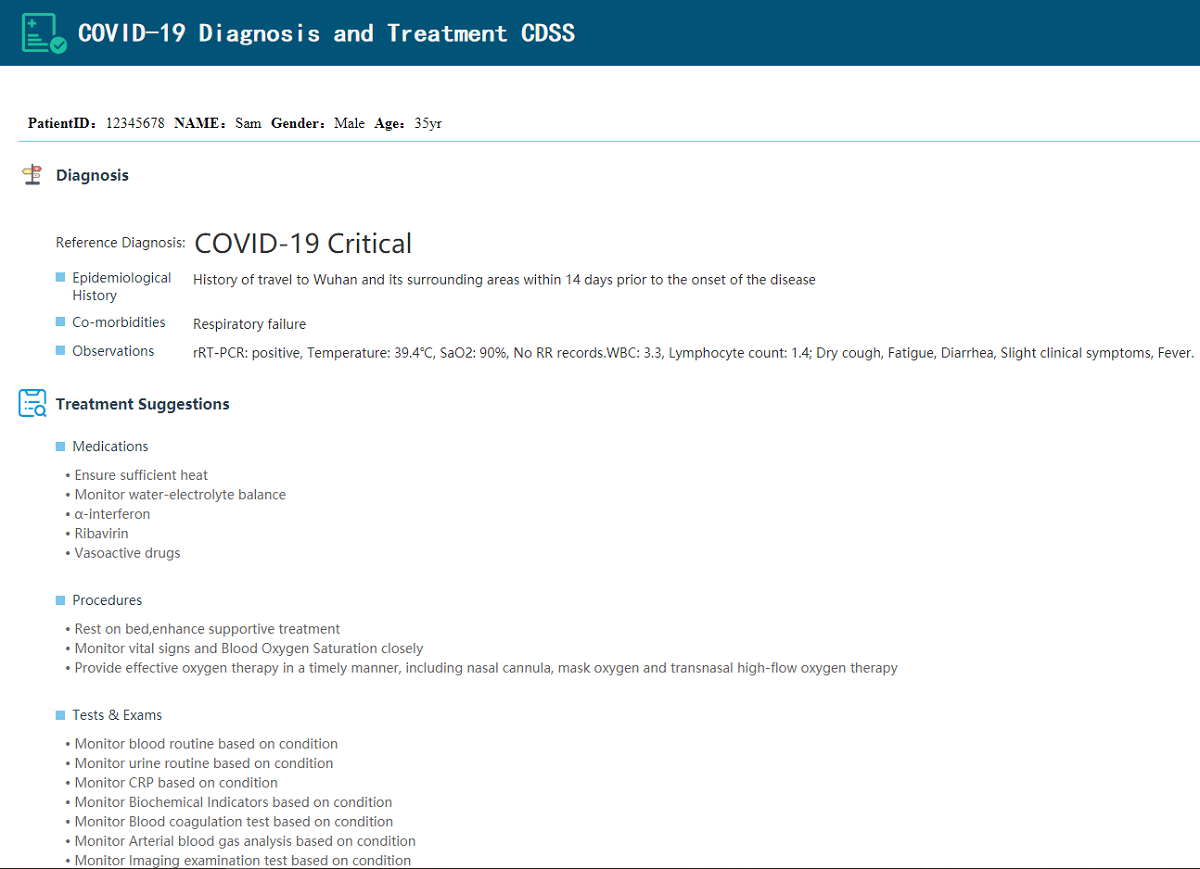
User interface of the prototype clinical decision support system application implementing the COVID-19 computer-interpretable guideline.

A prototype COVID-19 diagnosis and treatment CDSS was configured using the Tracebook platform [43]. The CDSS receives patient data from a web application program interface (API) and reasons with the COVID-19 CIG to provide evidence-based diagnosis and treatment suggestions. The user interface of the prototype is illustrated in Figure 3.

There are two blocks in the user interface: Diagnosis and Treatment Suggestions. The Diagnosis block is generated by the diagnosis, classification, and early warning rules. In the example in Figure 3, the patient is diagnosed with COVID-19, and the classification is critical. Data supporting the diagnosis are listed below the diagnosis. In the Treatment Suggestions block, medical suggestions, procedure suggestions, tests, and examination suggestions are provided according to the patient’s specific situation.

The CIG in both GDL and Drools language was validated by the published patient case report. The patient was diagnosed with critical COVID-19, and a detailed history was available along with vital signs, symptoms, image examinations, laboratory tests, and medication prescriptions. The diagnosis and treatment suggestions fit both the diagnosis and treatment plan in the case report and the Chinese COVID-19 Diagnosis and Treatment Plan. A detailed validation test report, including input and output data, is provided in Multimedia Appendix 3.

## Discussion

### Principal Results

We described a rapid development and dissemination approach to establish CIGs and applied this approach to a COVID-19 CIG. The COVID-19 pandemic is a global crisis that requires worldwide contributions from every domain [8]. While medical researchers have been establishing efficient diagnostic measures and effective treatment methodologies, informaticists are obligated to accelerate the wide adoption of these valuable best practices [7]. The usual approach of CIG development does not specify the input and output of each key step; therefore, informaticists and clinical specialists must engage in intensive discussions to understand each other. In our approach, we hastened this process by formalizing and structuring the discussions and reducing ambiguity.

The rapid CIG development approach makes the maximum use of existing medical knowledge sources (ie, openEHR archetypes) and parallels the tasks of domain concept modeling and clinical algorithm editing to further accelerate the process. During this study, four informaticists, two software engineers, and one external medical specialist were able to interpret and model the COVID-19 guideline and develop a prototype system remotely in four weeks. The rapid development and dissemination approach worked well for developing the COVID-19 CIG.

Goud et al [25] proposed a parallel guideline development and formalization strategy that encourages guideline development teams and the CIG development team to work closely together. By applying this strategy, the quality of the guideline and the efficiency of development of the CIG can both be improved. However, we argue that this strategy is not applicable in the COVID-19 crisis because the guideline was developed by a temporal national committee that we are not able to work with. In fact, in most current cases, clinical guidelines are still published by authorized committees or societies without the participation of informaticists. Van Gorp et al [44] proposed a model-driven engineering approach to rapidly translate annotated guideline knowledge to decision support applications. However, the procedure of annotation was not specified in their study.

The openEHR approach also possesses several potential advantages for future implementation. The openEHR approach provides a standard information model (ie, an archetype) that enables sharing of data definitions among organizations so that the “curly braces problem” is avoided. Moreover, the output of GDL2 rules is built based on archetypes; therefore, it is theoretically sharable among organizations. Indeed, it is estimated that 58 healthcare providers in 14 countries are currently using openEHR solutions [45]. The openEHR-based techniques can be translated into standardized HL7 Fast Healthcare Interoperability Resources (FHIR) format, which can be adopted by more EHRs [46].

### Limitations

The Guideline Elements Model (GEM) Cutter is a tool for annotating guidelines [47,48]. Due to the urgent development requirement, we did not use the GEM Cutter for the guideline annotation and extraction. A combination of GEM and GDL will be used in our future work.

Another limitation of our study is that the efficiency was not measured and compared with that of the usual approach. Because our primary goal was to rapidly develop and share a COVID-19 CIG, a comparison with other approaches was not performed. Moreover, for practical reasons, it is difficult to measure the exact time spent by each participant on each step. While developing this CIG, the researchers were locked down at home and working remotely. It is difficult to count the exact hours spent by each person because some of the researchers were using their spare time to perform this work, and working from home unavoidably scattered their working time.

For our prototype application, we did not use GDL as the execution language. There are three reasons for this. First, to the best of our knowledge, no dedicated execution engine for GDL2 is currently publicly available on the internet. Second, we did not manage to represent a time serial in GDL2 (eg, the last three nucleic acid tests were all negative); therefore, additional rules for data preprocessing in other languages were required. Last, there is a gap between the output of GDL2 rules and the actual requirements of the application. Thus, we used an open-source rule engine, Drools, instead. The GDL rules were manually translated to Drools rules with a set of predefined mapping rules. However, we believe that non-openEHR users can benefit from the object data model and Drools rules.

The rapid development and dissemination approach for CIGs has only been tested in the COVID-19 case. Although it worked well for our case, more tests are needed to determine its genericity. The COVID-19 guideline has been validated with a published patient case report. However, the clinical rules have not yet been applied to daily practice. When implementing these rules, it is likely that additional fine-tuning will be required to fit the local medical cultures and workflows of different health care providers.

### Conclusions

A CIG for COVID-19 can help caregivers provide evidence-based diagnosis and treatment to patients with COVID-19 to improve the quality of care. As yet, no such CIG exists due to the difficulty of rapid development. In this paper, we proposed a rapid development and dissemination approach for CIGs and developed a COVID-19 guideline by applying this approach. We hope that the COVID-19 CIG that we developed can help clinical information system vendors and care providers build their own CDSSs for COVID-19. Further, we hope that our approach can help other informaticists rapidly develop their own CIGs and share them globally in the future.

## Appendix

Multimedia Appendix 1 Segmented and structured Chinese COVID-19 guideline.

Multimedia Appendix 2 Extracted data items and mapping between archetypes and object data models.

Multimedia Appendix 3 Computer-interpretable guideline validation test report.

## Abbreviations

ADL: archetype definition language
API: application program interface
CDSS: clinical decision support system
CIG: computer-interpretable guideline
CKM: Clinical Knowledge Manager
EMR: electronic medical record
FHIR: Fast Healthcare Interoperability Resources
GDL: Guideline Definition Language
GEM: Guideline Elements Model
NHC: National Health Commission of the People’s Republic of China
