# Supplementary material for: A Computer-Interpretable Guideline for COVID-19: Rapid Development and Dissemination
Source: JMIR Med Inform. 2020 Oct 1;8(10):e21628. doi: 10.2196/21628 (PMC7546731; doi:10.2196/21628)
Supplement: Multimedia Appendix 3 [file medinform_v8i10e21628_app3.doc]

# Data source：First Case of 2019 Novel Coronavirus in the United States

## 1、The third day since admission

### Input

public Patient Init_P1_Day5(){
 //设置时间
 Date now = new Date();
 Date date1;
 long oneDayTime = 24*60*60*1000;
 long time = 4*60*60*1000;
 date1 = new Date(now.getTime()-oneDayTime);
 //设置流行病学史
 //发病前14天内有武汉市及周边地区的旅行史
 MedicalRecord medicalRecord1 = new MedicalRecord();
 medicalRecord1.setType("流行病学史");
 medicalRecord1.setText("Travel history of Wuhan and its surrounding areas within 14 days before illness onset");
 medicalRecord1.setDateTime(date1);
 patient.getMedicalRecordList().add(medicalRecord1);
// //症状
 Symptom symptom3 = new Symptom();
 symptom3.setText("Dry cough");symptom3.setReportDateTime(date1);patient.getSymptomList().add(symptom3);
 Symptom symptom4 = new Symptom();
 symptom4.setText("Fatigue");symptom4.setReportDateTime(date1);patient.getSymptomList().add(symptom4);
 Symptom symptom9 = new Symptom();
 symptom9.setText("Diarrhea");symptom9.setReportDateTime(date1);patient.getSymptomList().add(symptom9);
 Symptom symptom10 = new Symptom();
 symptom10.setText("Slight clinical symptoms");symptom10.setReportDateTime(date1);patient.getSymptomList().add(symptom10);
// 指氧饱和度
 PhysicalSign physicalSign_SpO2 = new PhysicalSign();
 physicalSign_SpO2.setItemName("SaO2");physicalSign_SpO2.setItemCode("SaO2");physicalSign_SpO2.setValue("90");
 physicalSign_SpO2.setUnit("%");physicalSign_SpO2.setMeasureDateTime(date1);
 patient.getPhysicalSignList().add(physicalSign_SpO2);
// 血压
 PhysicalSign physicalSign_BloodPressure = new PhysicalSign();
 physicalSign_BloodPressure.setItemName("Blood pressure");physicalSign_BloodPressure.setItemCode("Blood pressure");physicalSign_BloodPressure.setValue("134/87");
 physicalSign_BloodPressure.setUnit("mmHg");physicalSign_BloodPressure.setMeasureDateTime(date1);
 patient.getPhysicalSignList().add(physicalSign_BloodPressure);
// 脉搏
 PhysicalSign physicalSign_Pulse = new PhysicalSign();
 physicalSign_Pulse.setItemName("Pulse");physicalSign_Pulse.setItemCode("Pulse");physicalSign_Pulse.setValue("110");
 physicalSign_Pulse.setUnit("beats/min");physicalSign_Pulse.setMeasureDateTime(date1);
 patient.getPhysicalSignList().add(physicalSign_Pulse);
 //设置体温
 PhysicalSign physicalSign_T1 = new PhysicalSign();
 physicalSign_T1.setItemName("Body_temperature");physicalSign_T1.setItemCode("Body_temperature");physicalSign_T1.setValue("39.4");physicalSign_T1.setUnit("℃");
 physicalSign_T1.setMeasureDateTime(new Date(now.getTime()-time));
 patient.getPhysicalSignList().add(physicalSign_T1);
 //实验室检查
 LabTestResult PCR1 = new LabTestResult();
 PCR1.setItemName("rRT-PCR");PCR1.setResult("positive");PCR1.setUnit("");PCR1.setReferenceValue("");
 PCR1.setPerformDept("laboratory");PCR1.setStatus("Executed");PCR1.setRequestDept("comprehensive ICU");PCR1.setReportDateTime(date1);
 patient.getLabTestResultList().add(PCR1);
 LabTestResult WBC = new LabTestResult();
 WBC.setItemName("WBC");WBC.setResult("3.3");WBC.setUnit("");WBC.setReferenceValue("3.8-11");
 WBC.setPerformDept("laboratory");WBC.setStatus("Executed");WBC.setRequestDept("comprehensive ICU");WBC.setReportDateTime(date1);
 patient.getLabTestResultList().add(WBC);
 LabTestResult LBXB = new LabTestResult();
 LBXB.setItemName("Lymphocyte count");LBXB.setResult("1.4");LBXB.setUnit("");LBXB.setReferenceValue("1.0-3.9");
 LBXB.setPerformDept("laboratory");LBXB.setStatus("Executed");LBXB.setRequestDept("comprehensive ICU");LBXB.setReportDateTime(date1);
 patient.getLabTestResultList().add(LBXB);
 //医嘱
 //影像学检查
 ImgExamResult imgExamResult1 = new ImgExamResult();
 imgExamResult1.setDescription("Imaging showed no signs of pneumonia");imgExamResult1.setItemName("Chest CT");
 imgExamResult1.setReportDateTime(date1);patient.getImgExamResultList().add(imgExamResult1);
 //诊断
 PastHistory pastHistory = new PastHistory();

 Diagnosis diagnosis1 = new Diagnosis();
 diagnosis1.setItemName("Respiratory failure");diagnosis1.setCategory("Comorbities");
 pastHistory.getItems().add(diagnosis1);
 patient.getPastHistoryList().add(pastHistory);
 return patient;
 }

public PatientInfo Init_PInfo_Adult(){
 PatientInfo patientInfo = new PatientInfo();
 //病人基本信息
 patientInfo.setSex("Male");
 patientInfo.setName("Sam");
 patientInfo.setBloodType(new ArrayList<>(Arrays.*asList*("negative", "sd")));
 patientInfo.setAddress("Dream Land");
 SimpleDateFormat dateformat1 = new SimpleDateFormat("yyyy-MM-dd HH:mm:ss");
// SimpleDateFormat dateFormat2 = new SimpleDateFormat("yyyy-MM-dd HH:mm:ss");
 try {
 Date date = dateformat1.parse("2000-02-21 00:00:00");
 patientInfo.setDateOfBirth(date);
 }catch (Exception e){
// System.out.println(e);
 }
 System.*out*.println(dateformat1.format(patientInfo.getDateOfBirth()));
 patientInfo.setMaritalStatus("Married");
 patientInfo.setOccupation("farmer");
 patientInfo.setPhone("13866243621");
 patientInfo.setPatientId("123456");
 return patientInfo;
 }

### Output

{

    "code": 0,

    "serial": 0,

    "elapse": "192ms",

    "error": {

        "summary": "",

        "detail": ""

    },

    "data": {

        "patientInformation": {

            "admissionID": "",

            "patientID": "",

            "patientName": ""

        },

        "processes": [

            {

                "caseID": "",

                "processID": "GuidelineOutput",

                "processName": "GuidelineOutput",

                "scenarios": [

                    {

                        "groupedItems": null,

                        "inputData": null,

                        "isActive": "false",

                        "isScenarioGroup": "false",

                        "layout": null,

                        "note": null,

                        "performedTime": "2020-03-11 18:10:01",

                        "performers": null,

                        "postSubmittionRuleID": null,

                        "preloadRuleID": null,

                        "problems": [

                            {

                                "problemID": "Diagnosis",

                                "problemName": "Diagnosis",

                                "tasks": [

                                    {

                                        "assistantRuleID": null,

                                        "description": {

                                            "formatTexts": [

                                                {

                                                    "extension": null,

                                                    "type": 0,

                                                    "value": "Reference Diagnosis:"

                                                },

                                                {

                                                    "extension": null,

                                                    "type": 1,

                                                    "value": "COVID-19 Critical"

                                                }

                                            ]

                                        },

                                        "explanation": [

                                            {

                                                "formatTexts": [

                                                    {

                                                        "extension": null,

                                                        "type": 2,

                                                        "value": "Epidemiological History"

                                                    },

                                                    {

                                                        "extension": null,

                                                        "type": 0,

                                                        "value": "Travel history of Wuhan and its surrounding areas within 14 days before illness onset"

                                                    }

                                                ]

                                            },

                                            {

                                                "formatTexts": [

                                                    {

                                                        "extension": null,

                                                        "type": 2,

                                                        "value": "Co-morbidities"

                                                    },

                                                    {

                                                        "extension": null,

                                                        "type": 0,

                                                        "value": "Respiratory failure"

                                                    }

                                                ]

                                            },

                                            {

                                                "formatTexts": [

                                                    {

                                                        "extension": null,

                                                        "type": 2,

                                                        "value": "Observations"

                                                    },

                                                    {

                                                        "extension": null,

                                                        "type": 0,

                                                        "value": "rRT-PCR: positive, "

                                                    },

                                                    {

                                                        "extension": null,

                                                        "type": 0,

                                                        "value": "Temperature: 39.4℃, "

                                                    },

                                                    {

                                                        "extension": null,

                                                        "type": 0,

                                                        "value": "SaO2: 90%, "

                                                    },

                                                    {

                                                        "extension": null,

                                                        "type": 0,

                                                        "value": "No RR records."

                                                    },

                                                    {

                                                        "extension": null,

                                                        "type": 0,

                                                        "value": "WBC: 3.3, "

                                                    },

                                                    {

                                                        "extension": null,

                                                        "type": 0,

                                                        "value": "Lymphocyte count: 1.4; "

                                                    },

                                                    {

                                                        "extension": null,

                                                        "type": 0,

                                                        "value": "Dry cough, "

                                                    },

                                                    {

                                                        "extension": null,

                                                        "type": 0,

                                                        "value": "Fatigue, "

                                                    },

                                                    {

                                                        "extension": null,

                                                        "type": 0,

                                                        "value": "Diarrhea, "

                                                    },

                                                    {

                                                        "extension": null,

                                                        "type": 0,

                                                        "value": "Slight clinical symptoms, "

                                                    },

                                                    {

                                                        "extension": null,

                                                        "type": 0,

                                                        "value": "Fever."

                                                    }

                                                ]

                                            }

                                        ],

                                        "mandatory": true,

                                        "note": null,

                                        "postEffectID": null,

                                        "preConditionID": null,

                                        "result": null,

                                        "taskID": null,

                                        "taskInstanceID": null,

                                        "taskName": null,

                                        "type": null

                                    }

                                ]

                            },

                            {

                                "problemID": "Treatment Suggestions",

                                "problemName": "Treatment Suggestions",

                                "tasks": [

                                    {

                                        "assistantRuleID": null,

                                        "description": {

                                            "formatTexts": [

                                                {

                                                    "extension": null,

                                                    "type": 2,

                                                    "value": "Procedures"

                                                }

                                            ]

                                        },

                                        "explanation": [

                                            {

                                                "formatTexts": [

                                                    {

                                                        "extension": null,

                                                        "type": 8,

                                                        "value": "Admitted to hospital"

                                                    }

                                                ]

                                            }

                                        ],

                                        "mandatory": false,

                                        "note": null,

                                        "postEffectID": null,

                                        "preConditionID": null,

                                        "result": "false",

                                        "taskID": null,

                                        "taskInstanceID": null,

                                        "taskName": null,

                                        "type": "false"

                                    }

                                ]

                            }

                        ],

                        "refScenario": null,

                        "role": null,

                        "scenarioID": "OUTPATIENT",

                        "scenarioInstanceID": null,

                        "scenarioName": "OUTPATIENT",

                        "status": null

                    },

                    {

                        "groupedItems": null,

                        "inputData": null,

                        "isActive": "false",

                        "isScenarioGroup": "false",

                        "layout": null,

                        "note": null,

                        "performedTime": "2020-03-11 18:10:01",

                        "performers": null,

                        "postSubmittionRuleID": null,

                        "preloadRuleID": null,

                        "problems": [

                            {

                                "problemID": "Diagnosis",

                                "problemName": "Diagnosis",

                                "tasks": [

                                    {

                                        "assistantRuleID": null,

                                        "description": {

                                            "formatTexts": [

                                                {

                                                    "extension": null,

                                                    "type": 0,

                                                    "value": "Reference Diagnosis:"

                                                },

                                                {

                                                    "extension": null,

                                                    "type": 1,

                                                    "value": "COVID-19 Critical"

                                                }

                                            ]

                                        },

                                        "explanation": [

                                            {

                                                "formatTexts": [

                                                    {

                                                        "extension": null,

                                                        "type": 2,

                                                        "value": "Epidemiological History"

                                                    },

                                                    {

                                                        "extension": null,

                                                        "type": 0,

                                                        "value": "Travel history of Wuhan and its surrounding areas within 14 days before illness onset"

                                                    }

                                                ]

                                            },

                                            {

                                                "formatTexts": [

                                                    {

                                                        "extension": null,

                                                        "type": 2,

                                                        "value": "Co-morbidities"

                                                    },

                                                    {

                                                        "extension": null,

                                                        "type": 0,

                                                        "value": "Respiratory failure"

                                                    }

                                                ]

                                            },

                                            {

                                                "formatTexts": [

                                                    {

                                                        "extension": null,

                                                        "type": 2,

                                                        "value": "Observations"

                                                    },

                                                    {

                                                        "extension": null,

                                                        "type": 0,

                                                        "value": "rRT-PCR: positive, "

                                                    },

                                                    {

                                                        "extension": null,

                                                        "type": 0,

                                                        "value": "Temperature: 39.4℃, "

                                                    },

                                                    {

                                                        "extension": null,

                                                        "type": 0,

                                                        "value": "SaO2: 90%, "

                                                    },

                                                    {

                                                        "extension": null,

                                                        "type": 0,

                                                        "value": "No RR records."

                                                    },

                                                    {

                                                        "extension": null,

                                                        "type": 0,

                                                        "value": "WBC: 3.3, "

                                                    },

                                                    {

                                                        "extension": null,

                                                        "type": 0,

                                                        "value": "Lymphocyte count: 1.4; "

                                                    },

                                                    {

                                                        "extension": null,

                                                        "type": 0,

                                                        "value": "Dry cough, "

                                                    },

                                                    {

                                                        "extension": null,

                                                        "type": 0,

                                                        "value": "Fatigue, "

                                                    },

                                                    {

                                                        "extension": null,

                                                        "type": 0,

                                                        "value": "Diarrhea, "

                                                    },

                                                    {

                                                        "extension": null,

                                                        "type": 0,

                                                        "value": "Slight clinical symptoms, "

                                                    },

                                                    {

                                                        "extension": null,

                                                        "type": 0,

                                                        "value": "Fever."

                                                    }

                                                ]

                                            }

                                        ],

                                        "mandatory": true,

                                        "note": null,

                                        "postEffectID": null,

                                        "preConditionID": null,

                                        "result": null,

                                        "taskID": null,

                                        "taskInstanceID": null,

                                        "taskName": null,

                                        "type": null

                                    }

                                ]

                            },

                            {

                                "problemID": "Treatment Suggestions",

                                "problemName": "Treatment Suggestions",

                                "tasks": [

                                    {

                                        "assistantRuleID": null,

                                        "description": {

                                            "formatTexts": [

                                                {

                                                    "extension": null,

                                                    "type": 2,

                                                    "value": "Medications"

                                                }

                                            ]

                                        },

                                        "explanation": [

                                            {

                                                "formatTexts": [

                                                    {

                                                        "extension": null,

                                                        "type": 8,

                                                        "value": "Guaranteed sufficient heat"

                                                    },

                                                    {

                                                        "extension": null,

                                                        "type": 8,

                                                        "value": "Pay attention to water-electrolyte balance"

                                                    },

                                                    {

                                                        "extension": null,

                                                        "type": 8,

                                                        "value": "α-interferon"

                                                    },

                                                    {

                                                        "extension": null,

                                                        "type": 8,

                                                        "value": "Ribavirin"

                                                    },

                                                    {

                                                        "extension": null,

                                                        "type": 8,

                                                        "value": "Vasoactive drugs"

                                                    },

                                                    {

                                                        "extension": null,

                                                        "type": 8,

                                                        "value": "Recommended Chinese patent medicine-Foundation formula：麻黄9g、炙甘草6g、杏仁9g、生石膏15〜30g（先煎）、桂枝9g、泽泻9g、猪苓9g、白术9g、茯苓15g、柴胡16g、黄芩6g、姜半夏9g、生姜9g、紫苑9g、冬花9g、射干9g、细辛6g、山药12g、枳实6g、陈皮6g、着香9g。\n服法：传统中药饮片，水煎服。每天一付，早晚两次（饭后四十分钟），温服，三付一个疗程。"

                                                    }

                                                ]

                                            }

                                        ],

                                        "mandatory": false,

                                        "note": null,

                                        "postEffectID": null,

                                        "preConditionID": null,

                                        "result": "false",

                                        "taskID": null,

                                        "taskInstanceID": null,

                                        "taskName": null,

                                        "type": "false"

                                    },

                                    {

                                        "assistantRuleID": null,

                                        "description": {

                                            "formatTexts": [

                                                {

                                                    "extension": null,

                                                    "type": 2,

                                                    "value": "Procedures"

                                                }

                                            ]

                                        },

                                        "explanation": [

                                            {

                                                "formatTexts": [

                                                    {

                                                        "extension": null,

                                                        "type": 8,

                                                        "value": "Rest on bed,enhance supportive treatment"

                                                    },

                                                    {

                                                        "extension": null,

                                                        "type": 8,

                                                        "value": "Monitor vital signs and Blood Oxygen Saturation closely"

                                                    },

                                                    {

                                                        "extension": null,

                                                        "type": 8,

                                                        "value": "Provide effective oxygen therapy in a timely manner, including nasal cannula, mask oxygen and transnasal high-flow oxygen therapy"

                                                    },

                                                    {

                                                        "extension": null,

                                                        "type": 8,

                                                        "value": "Admitted to ICU"

                                                    }

                                                ]

                                            }

                                        ],

                                        "mandatory": false,

                                        "note": null,

                                        "postEffectID": null,

                                        "preConditionID": null,

                                        "result": "false",

                                        "taskID": null,

                                        "taskInstanceID": null,

                                        "taskName": null,

                                        "type": "false"

                                    },

                                    {

                                        "assistantRuleID": null,

                                        "description": {

                                            "formatTexts": [

                                                {

                                                    "extension": null,

                                                    "type": 2,

                                                    "value": "Tests & Exams"

                                                }

                                            ]

                                        },

                                        "explanation": [

                                            {

                                                "formatTexts": [

                                                    {

                                                        "extension": null,

                                                        "type": 8,

                                                        "value": "Monitor blood routine based on condition"

                                                    },

                                                    {

                                                        "extension": null,

                                                        "type": 8,

                                                        "value": "Monitor urine routine based on condition"

                                                    },

                                                    {

                                                        "extension": null,

                                                        "type": 8,

                                                        "value": "Monitor CRP based on condition"

                                                    },

                                                    {

                                                        "extension": null,

                                                        "type": 8,

                                                        "value": "Monitor Biochemical Indicators based on condition"

                                                    },

                                                    {

                                                        "extension": null,

                                                        "type": 8,

                                                        "value": "Monitor Blood coagulation test based on condition"

                                                    },

                                                    {

                                                        "extension": null,

                                                        "type": 8,

                                                        "value": "Monitor Arterial blood gas analysis based on condition"

                                                    },

                                                    {

                                                        "extension": null,

                                                        "type": 8,

                                                        "value": "Monitor Imaging examination test based on condition"

                                                    },

                                                    {

                                                        "extension": null,

                                                        "type": 8,

                                                        "value": "Monitor Cytokine detection based on condition"

                                                    },

                                                    {

                                                        "extension": null,

                                                        "type": 8,

                                                        "value": "Hemodynamic monitoring if necessary"

                                                    }

                                                ]

                                            }

                                        ],

                                        "mandatory": false,

                                        "note": null,

                                        "postEffectID": null,

                                        "preConditionID": null,

                                        "result": "false",

                                        "taskID": null,

                                        "taskInstanceID": null,

                                        "taskName": null,

                                        "type": "false"

                                    }

                                ]

                            }

                        ],

                        "refScenario": null,

                        "role": null,

                        "scenarioID": "INPATIENT",

                        "scenarioInstanceID": null,

                        "scenarioName": "INPATIENT",

                        "status": null

                    },

                    {

                        "groupedItems": null,

                        "inputData": null,

                        "isActive": "false",

                        "isScenarioGroup": "false",

                        "layout": null,

                        "note": null,

                        "performedTime": "2020-03-11 18:10:01",

                        "performers": null,

                        "postSubmittionRuleID": null,

                        "preloadRuleID": null,

                        "problems": [

                            {

                                "problemID": "Diagnosis",

                                "problemName": "Diagnosis",

                                "tasks": [

                                    {

                                        "assistantRuleID": null,

                                        "description": {

                                            "formatTexts": [

                                                {

                                                    "extension": null,

                                                    "type": 0,

                                                    "value": "Reference Diagnosis:"

                                                },

                                                {

                                                    "extension": null,

                                                    "type": 1,

                                                    "value": "COVID-19 Critical"

                                                }

                                            ]

                                        },

                                        "explanation": [

                                            {

                                                "formatTexts": [

                                                    {

                                                        "extension": null,

                                                        "type": 2,

                                                        "value": "Epidemiological History"

                                                    },

                                                    {

                                                        "extension": null,

                                                        "type": 0,

                                                        "value": "Travel history of Wuhan and its surrounding areas within 14 days before illness onset"

                                                    }

                                                ]

                                            },

                                            {

                                                "formatTexts": [

                                                    {

                                                        "extension": null,

                                                        "type": 2,

                                                        "value": "Co-morbidities"

                                                    },

                                                    {

                                                        "extension": null,

                                                        "type": 0,

                                                        "value": "Respiratory failure"

                                                    }

                                                ]

                                            },

                                            {

                                                "formatTexts": [

                                                    {

                                                        "extension": null,

                                                        "type": 2,

                                                        "value": "Observations"

                                                    },

                                                    {

                                                        "extension": null,

                                                        "type": 0,

                                                        "value": "rRT-PCR: positive, "

                                                    },

                                                    {

                                                        "extension": null,

                                                        "type": 0,

                                                        "value": "Temperature: 39.4℃, "

                                                    },

                                                    {

                                                        "extension": null,

                                                        "type": 0,

                                                        "value": "SaO2: 90%, "

                                                    },

                                                    {

                                                        "extension": null,

                                                        "type": 0,

                                                        "value": "No RR records."

                                                    },

                                                    {

                                                        "extension": null,

                                                        "type": 0,

                                                        "value": "WBC: 3.3, "

                                                    },

                                                    {

                                                        "extension": null,

                                                        "type": 0,

                                                        "value": "Lymphocyte count: 1.4; "

                                                    },

                                                    {

                                                        "extension": null,

                                                        "type": 0,

                                                        "value": "Dry cough, "

                                                    },

                                                    {

                                                        "extension": null,

                                                        "type": 0,

                                                        "value": "Fatigue, "

                                                    },

                                                    {

                                                        "extension": null,

                                                        "type": 0,

                                                        "value": "Diarrhea, "

                                                    },

                                                    {

                                                        "extension": null,

                                                        "type": 0,

                                                        "value": "Slight clinical symptoms, "

                                                    },

                                                    {

                                                        "extension": null,

                                                        "type": 0,

                                                        "value": "Fever."

                                                    }

                                                ]

                                            }

                                        ],

                                        "mandatory": true,

                                        "note": null,

                                        "postEffectID": null,

                                        "preConditionID": null,

                                        "result": null,

                                        "taskID": null,

                                        "taskInstanceID": null,

                                        "taskName": null,

                                        "type": null

                                    }

                                ]

                            },

                            {

                                "problemID": "Treatment Suggestions",

                                "problemName": "Treatment Suggestions",

                                "tasks": [

                                    {

                                        "assistantRuleID": null,

                                        "description": {

                                            "formatTexts": [

                                                {

                                                    "extension": null,

                                                    "type": 2,

                                                    "value": "Medications"

                                                }

                                            ]

                                        },

                                        "explanation": [

                                            {

                                                "formatTexts": [

                                                    {

                                                        "extension": null,

                                                        "type": 8,

                                                        "value": "Guaranteed sufficient heat"

                                                    },

                                                    {

                                                        "extension": null,

                                                        "type": 8,

                                                        "value": "Pay attention to water-electrolyte balance"

                                                    },

                                                    {

                                                        "extension": null,

                                                        "type": 8,

                                                        "value": "α-interferon"

                                                    },

                                                    {

                                                        "extension": null,

                                                        "type": 8,

                                                        "value": "Ribavirin"

                                                    },

                                                    {

                                                        "extension": null,

                                                        "type": 8,

                                                        "value": "Vasoactive drugs"

                                                    },

                                                    {

                                                        "extension": null,

                                                        "type": 8,

                                                        "value": "Recommended Chinese patent medicine-Foundation formula：麻黄9g、炙甘草6g、杏仁9g、生石膏15〜30g（先煎）、桂枝9g、泽泻9g、猪苓9g、白术9g、茯苓15g、柴胡16g、黄芩6g、姜半夏9g、生姜9g、紫苑9g、冬花9g、射干9g、细辛6g、山药12g、枳实6g、陈皮6g、着香9g。\n服法：传统中药饮片，水煎服。每天一付，早晚两次（饭后四十分钟），温服，三付一个疗程。"

                                                    }

                                                ]

                                            }

                                        ],

                                        "mandatory": false,

                                        "note": null,

                                        "postEffectID": null,

                                        "preConditionID": null,

                                        "result": "false",

                                        "taskID": null,

                                        "taskInstanceID": null,

                                        "taskName": null,

                                        "type": "false"

                                    },

                                    {

                                        "assistantRuleID": null,

                                        "description": {

                                            "formatTexts": [

                                                {

                                                    "extension": null,

                                                    "type": 2,

                                                    "value": "Procedures"

                                                }

                                            ]

                                        },

                                        "explanation": [

                                            {

                                                "formatTexts": [

                                                    {

                                                        "extension": null,

                                                        "type": 8,

                                                        "value": "Rest on bed,enhance supportive treatment"

                                                    },

                                                    {

                                                        "extension": null,

                                                        "type": 8,

                                                        "value": "Monitor vital signs and Blood Oxygen Saturation closely"

                                                    },

                                                    {

                                                        "extension": null,

                                                        "type": 8,

                                                        "value": "Provide effective oxygen therapy in a timely manner, including nasal cannula, mask oxygen and transnasal high-flow oxygen therapy"

                                                    }

                                                ]

                                            }

                                        ],

                                        "mandatory": false,

                                        "note": null,

                                        "postEffectID": null,

                                        "preConditionID": null,

                                        "result": "false",

                                        "taskID": null,

                                        "taskInstanceID": null,

                                        "taskName": null,

                                        "type": "false"

                                    },

                                    {

                                        "assistantRuleID": null,

                                        "description": {

                                            "formatTexts": [

                                                {

                                                    "extension": null,

                                                    "type": 2,

                                                    "value": "Tests & Exams"

                                                }

                                            ]

                                        },

                                        "explanation": [

                                            {

                                                "formatTexts": [

                                                    {

                                                        "extension": null,

                                                        "type": 8,

                                                        "value": "Monitor blood routine based on condition"

                                                    },

                                                    {

                                                        "extension": null,

                                                        "type": 8,

                                                        "value": "Monitor urine routine based on condition"

                                                    },

                                                    {

                                                        "extension": null,

                                                        "type": 8,

                                                        "value": "Monitor CRP based on condition"

                                                    },

                                                    {

                                                        "extension": null,

                                                        "type": 8,

                                                        "value": "Monitor Biochemical Indicators based on condition"

                                                    },

                                                    {

                                                        "extension": null,

                                                        "type": 8,

                                                        "value": "Monitor Blood coagulation test based on condition"

                                                    },

                                                    {

                                                        "extension": null,

                                                        "type": 8,

                                                        "value": "Monitor Arterial blood gas analysis based on condition"

                                                    },

                                                    {

                                                        "extension": null,

                                                        "type": 8,

                                                        "value": "Monitor Imaging examination test based on condition"

                                                    },

                                                    {

                                                        "extension": null,

                                                        "type": 8,

                                                        "value": "Monitor Cytokine detection based on condition"

                                                    },

                                                    {

                                                        "extension": null,

                                                        "type": 8,

                                                        "value": "Hemodynamic monitoring if necessary"

                                                    }

                                                ]

                                            }

                                        ],

                                        "mandatory": false,

                                        "note": null,

                                        "postEffectID": null,

                                        "preConditionID": null,

                                        "result": "false",

                                        "taskID": null,

                                        "taskInstanceID": null,

                                        "taskName": null,

                                        "type": "false"

                                    }

                                ]

                            }

                        ],

                        "refScenario": null,

                        "role": null,

                        "scenarioID": "ICU",

                        "scenarioInstanceID": null,

                        "scenarioName": "ICU",

                        "status": null

                    }

                ],

                "status": ""

            }

        ]

    }

}
